# Epidemiological profile of patients with preterm premature rupture of membranes at a tertiary hospital in São Paulo, Brazil

**DOI:** 10.6061/clinics/2019/e1231

**Published:** 2019-10-14

**Authors:** Marco Aurélio Knippel Galletta, Roberto Eduardo Bittar, Isabela Agra, Eliane Cerqueira Leite Guerra, Rossana Pulcineli Vieira Francisco, Marcelo Zugaib

**Affiliations:** Disciplina de Obstetricia, Departamento de Obstetricia e Ginecologia, Faculdade de Medicina FMUSP, Universidade de Sao Paulo, Sao Paulo, SP, BR

**Keywords:** Fetal Membranes, Premature Rupture, Pregnancy Outcome, Pregnancy Complications, Obstetric Labor, Premature, Infant, Newborn, Perinatal Death

## Abstract

**OBJECTIVE:**

To perform a descriptive analysis of preterm premature rupture of membranes (PPROM) cases attended in a tertiary hospital.

**METHOD:**

Retrospective analysis of medical records and laboratory tests of patients admitted to a Brazilian tertiary hospital between 2006 and 2011, with a confirmed diagnosis of PPROM and gestational age (GA) at delivery <37 weeks.

**RESULTS:**

A total of 299 pregnant women were included in the study. Nine patients evolved to abortion, and 290 pregnant women remained for the final analysis. There was initial diagnostic doubt in 17.6% of the cases. The oligohydramnios rate [amniotic fluid index (AFI) <5] was 27.9% on admission. Chorioamnionitis was initially diagnosed in 10.8% of the patients and was retrospectively confirmed in 22.9% of the samples. The latency period had a mean of 9.1 days. The main reasons for interruption were premature labor (55.2%), GA ≥36 weeks (27.2%), and fetal distress (6.9%). The delivery method was cesarean section in 55% of cases. The mean birth weight was 2,124 grams, and 67% of the neonates had a low birth weight (<2500 g). The GA at delivery averaged 33.5 weeks. The stillbirth rate was 5.3%, and the early neonatal mortality rate was 5.6%. There were complications at delivery in 18% of mothers.

**CONCLUSION:**

In one of the few Brazilian reports on the epidemiological profile of PPROM, with GA until 37 weeks and intercurrences generally excluded from assessments (such as twinning and fetal malformations), there is a favorable evolution, with an acceptable rate of complications.

## INTRODUCTION

Premature rupture of membranes (PROM), defined as rupture of the amnion and chorion before labor, represents a serious public health problem when it occurs before the 37^th^ gestational week. The so-called preterm premature rupture of membranes (PPROM) affects approximately 2-3% of pregnant women (1), accounting for approximately one-third of premature births, with rates of 32.6% in the United States (2) and 28.7% in Brazil (3). According to some authors, PPROM is the most common intercurrence in prematurity (4).

PPROM generally presents a great dilemma for the attending physician, who must determine the best time for intervention by balancing the risks of prematurity on the one hand and the risks of intra-amniotic infection and intrauterine fetal death on the other. Despite great controversy regarding the optimal clinical conduct (5), approximately half of the infants of women with PPROM tend to be born within a week and face the risks inherent to prematurity (1).

This scenario is more dramatic in developing countries, where high rates of prematurity are associated with neonatal care conditions and vacancies in neonatal intensive care units that are less than ideal (6). Despite this high prevalence, and especially in Brazil, there are few scientific publications regarding PPROM, especially in terms of the epidemiology, management and complications of this obstetric pathology, and these publications lack a complete description of patients with late prematurity or cases of twins and fetal malformations (7-12). Against this background, we aimed to perform a detailed and critical analysis of our experiences with PPROM, considering its demographic, obstetric and neonatal characteristics and main complications, at a Brazilian tertiary service, with the intention of seeking more appropriate approaches in such cases.

## MATERIALS AND METHODS

We performed a retrospective analysis of the PPROM cases admitted to the Obstetrics Clinic of the Hospital das Clinicas of FMUSP, São Paulo, Brazil, between 2006 and 2011 that met the following inclusion criteria: a confirmed diagnosis of PPROM; gestational age (GA) at delivery <37 weeks; and childbirth data available. The patients were selected from the computer system used on the ward (Access MS), and medical records and laboratory tests were verified using the electronic platform of the Central Laboratory of Hospital das Clinicas.

The patients were treated according to the protocol of the Hospital das Clinicas of the FMUSP obstetrics clinic, which is based on a previously published protocol (13). This protocol includes expectant management up to 36 weeks and daily assessments of fetal vitality, including the quantification of the amount of amniotic fluid according to the technique of Phelan et al. (14) and monitoring of respiratory movements according to the technique of Manning et al. (15), as well as laboratory evaluations of leukocytes and C-reactive protein (CRP) every two days. Follow-up was continued until the 36^th^ week of pregnancy, at which time, gestation was interrupted. The pregnancy was interrupted before then only in cases of preterm labor (which was not inhibited), altered fetal vitality or chorioamnionitis. A diagnosis of chorioamnionitis was assumed in the presence of 2 simultaneous clinical or laboratory signs, including leukocytosis (>15,000/mm^3^) or a ≥20% increase in leukocytes or CRP. For the present study, we evaluated CRP in two ways: according to the laboratory cut-off value (>5 mg/L) and in according to the percentage increase compared with previous values. To determine the adequacy of the increase in birth weight, we used the American curve described by Alexander et al. (16). The data were arranged in a spreadsheet and analyzed using the statistical program IBM SPSS Statistics 20. Numerical variables are described as the means, medians, variations (minimum-maximum), interquartile distributions (25%-75%), and standard deviations (SDs). Categorical variables are described as frequencies, percentages and cut-off levels. To compare some variables with each other, we used the Mann-Whitney U Test, assuming a level of significance (α) of 5%.

### Ethical Aspects

The research project was approved by the department’s internal committee for the approval of research projects (CIAPP) and by the Research Projects Approval Committee (CAPPesq) of the Hospital das Clinicas da FMUSP, with Technical Advice Number 248040, dated 04/17/2013. It was registered in the Brazil Platform under the number CAAE 13978413.2.0000.0068. Because it was a retrospective research project, consent was not necessary; however, care was taken when handling the patients’ records, and the patients were not identified. There was no external funding, and the authors declare no conflict of interest in the publication.

## RESULTS

In the study period, we identified 418 cases of possible PPROM. The number of births that occurred in this same period was 4,669; therefore, a prevalence of 8.95% was calculated. Of the total deliveries, 2,606 were before the 37^th^ week. That is, among premature births, the PPROM rate was 16.04%. However, some of those PPROM cases were discharged without return, or the medical record was not found. Thus, the final analysis included data from 299 pregnant women who met the inclusion criteria. Their characteristics are presented in Tables 1 (numerical variables) and 2 (categorical variables).

Some risk factors typically related to the occurrence of PPROM could be identified, as follows: a history of previous preterm birth (17.3%); smoking (14%); urinary tract infection (13.7%); fetal malformation (13.4%); twinning (11.7%); preterm labor during the current pregnancy prior to PPROM (9.9%); diabetes (8.1%); previous PROM (6.6%); bleeding during the first half of pregnancy (5.8%); cervical incompetence (3.9%); and cerclage (4.3%). In addition, some clinical and/or obstetric comorbidities were present in 173 pregnant women (69.2%).

During their initial clinical presentation, these pregnant women presented typical complaints in 78.2% of the cases, and diagnostic doubt was present in 17.6% of the cases. In 83.3% of the patients, the diagnosis was confirmed by the visualization of amniotic fluid in the vaginal sac fundus. The phenol test was performed in 111 patients (37.1%); the results were positive in 100 (90.1%), negative in 6 (5.4%), and unclear in 5 (4.5%). Confirmation with vaginal buffer was necessary in 11 patients (3.9%). In 35 cases (12.7%), there was concomitant bleeding in the initial setting, making diagnosis difficult.

Vaginal cultures at admission were positive in the following proportions: Group B streptococcus in 20.2%; fungi in 9.3%; trichomonas in 1.85%; and chlamydia in 1.58% of patients. No patient was positive for gonococcus. The bacterioscopic examinations of vaginal secretions were altered, with Nugent ≥4, in 30% of the patients investigated.

In terms of the evolution of the condition, 9 patients (3.0%) progressed to abortion (IG<20 weeks), with a mean GA of 17.32 weeks (SD=1.596) at the time of PPROM and 18.09 (SD=1.852) at the time of elimination of the fetus, which weighed on average 191 grams (SD=100.42). The latency period was on average 6.12 days (SD=4.912). Of these 9 patients, 4 (44.4%) had a clinical diagnosis of chorioamnionitis. In the retrospective evaluation, 5 patients (55.5%) were definitively diagnosed with chorioamnionitis. Despite the small number of patients, there was a higher rate of chorioamnionitis in this subgroup than in the overall sample.

We excluded these nine patients from subsequent evaluations. The remaining 290 patients, who were included in the analyses described henceforth, evolved as follows.

As can be seen from the analysis in Table 3, during hospitalization, there was a progressive decrease in amniotic fluid, as well as increased leukocyte count and CRP. The final AFI was <5.0, indicating oligohydramnios, in 37.8% of the cases. There was a decrease in AFI during hospitalization in 72% of the cases, and the final AFI was lower than the penultimate value in 57% of the patients. There was severe oligohydramnios (AFI<3) at some point during hospitalization in 22.3% of the cases.

A total of 53 patients (23.66%) had leukocytosis (>15,000/mm^3^) at some point during hospitalization The CRP was greater than the reference value (<5) in the first evaluation in 63/90 pregnant women (21.6%) and, in the final evaluation, in 104/164 pregnant women (72.2%). CRP was higher at the last evaluation than at the first evaluation in 64.8% of the pregnant women, and this increase was greater than 20% in 54% of patients. When only the last two evaluations were considered, CRP increased in 70.1% of the patients, and this difference was greater than 20% in 50.7% of them. The lack of some data in pregnant women in relation to the Initial CRP was due to a shorter hospitalization time for these patients. Separating the groups without and with Initial CRP, a clear discrepancy is evident: 4.08 *vs*. 20.11 days, respectively, in average latency period and 5.9 *vs*. 16.24 days, respectively, of hospitalization on average (*p*=0.000). On the other hand, the mean birth weight was higher in the group without Initial CRP (2,232 *vs*. 1,978 g, *p*=0.013), as well as a higher mean GA at delivery (33.95 *vs*. 32.58 weeks; *p*=0.001), perhaps because patients of near GA would not benefit greatly from the CRP dosage. The same can be said for the cases without and with Initial AFI, which also had a lower average latency time (4.17 *vs*. 13.90 days, *p*=0.000) and a lower mean number of days of hospitalization (6.13 *vs*. 12, 02; *p*=0.000), but with similar GAs and birth weights.

There was a clinical diagnosis of chorioamnionitis (at least 2 clinical and/or laboratory criteria present at the same time) in 31 cases (10.8%). In comparison, histological chorioamnionitis was diagnosed through an examination of pathological anatomy in 71 cases (29%). Overall, the presence of chorioamnionitis was observed retrospectively in 55 cases (22.9%). That is, in 24 cases (8.2% of the total of the pregnant group of women and 43.6% of the cases with a final diagnosis of chorioamnionitis), the clinical diagnosis of chorioamnionitis was only confirmed later and was not initially noticed.

Antibiotics were prescribed before delivery for 61 patients (29.9%); the most common indications were Group B streptococcus (30 or 15.6%) and urinary tract infection (23 or 10.9%).

Antenatal corticosteroid therapy was used in only 21 patients (9.9%), with a mean GA of 29.94 weeks, ranging from 24.14 to 34 weeks. In two patients, corticosteroids were used more than once.

The period of hospitalization for these mothers varied from 1 to 133 days, with a concentration between 3 and 11 days (25% -75%), a mean of 9.12 days and a median of 5 days. Hospital admission was ≥7 days in 40.2% of the patients and was prolonged, ≥30 days, in only 5.4% of the sample. The latency period for the group as a whole ranged from 0 to 145 days; it was concentrated between 1 and 10 days (25-75%), with a mean of 9.17 and a median of 2 days. The period between diagnosis and delivery was ≥3 days in 48.5%, ≥7 days in 31.4% and ≥30 days in 7.9% of the sample.

**Figure 1:**
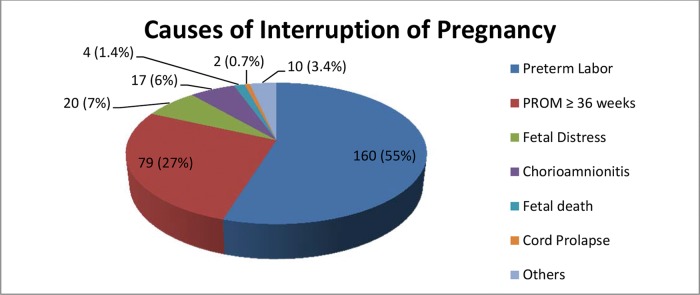
Distribution of causes of pregnancy interruption, in 290 pregnant women with PPROM. Sao Paulo, Brazil, 2006-2011.

Regarding delivery, as shown in Figure 1, more than half of the patients (160 or 55.2%) spontaneously went into preterm labor, which was the reason for the interruption of the pregnancy. More than a quarter of the pregnant women (79 or 27.2%) had their pregnancy interrupted because they had reached the 36th gestational week, in accordance with hospital protocol. Other major causes of discontinuation were fetal distress (20 cases or 6.9%), a diagnosis of chorioamnionitis (17 or 5.9%) and fetal death (4 or 1.4%). Less frequent causes of discontinuation were cord prolapse (2 cases or 0.7%), meconium in the amniotic fluid (2), abruptio placentae (1) and bleeding (1), among others, totaling 10 cases or 3.4%. Labor was induced on 47 occasions (16.4%), and misoprostol was used to prepare and ripen the cervix in 15 cases (5.2%).

The duration of labor for the sample as a whole ranged from 0 to 96 hours, with an average of 5.06 hours, a median of 4 hours, and the greatest distribution between 2 and 6 hours (25-75%). More than two-thirds of the patients (154 or 75.9%) had an identified labor duration ≤6 hours. A foul smell was present in 30 deliveries of the entire sample (11.6%).

**Figure 2:**
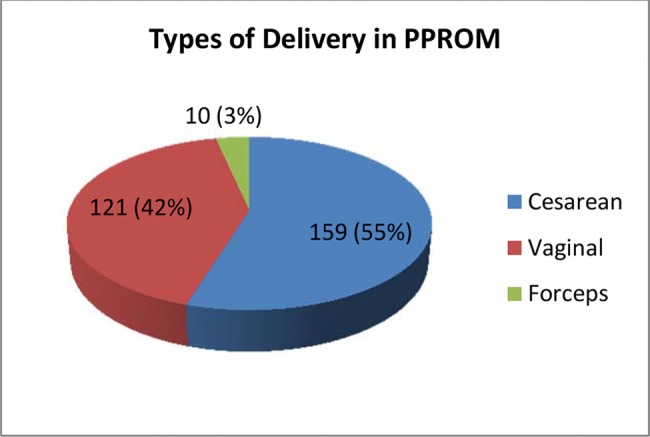
Distribution of types of delivery in 290 cases of PPROM, excluding abortions. Sao Paulo, Brazil, 2006-2011.

Regarding the type of delivery, the majority of the patients (159 or 55%) had a cesarean delivery, while 121 (42%) had a vaginal delivery, and only 10 (3%) required the use of forceps (Figure 2).

**Figure 3:**
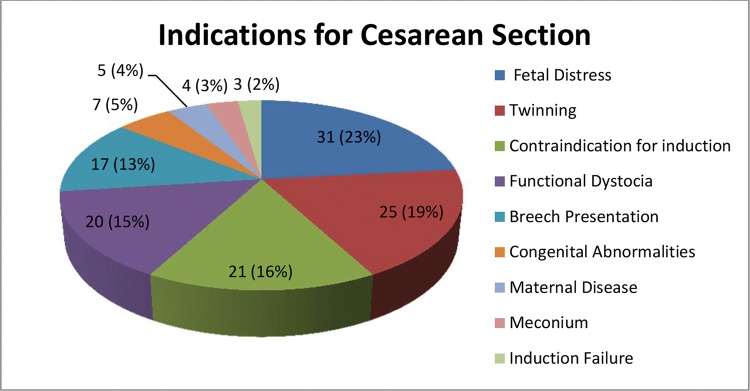
Distribution of indications for cesarean delivery in 159 pregnant women with PPROM. Sao Paulo, Brazil, 2006-2011.

As Figure 3 shows, the indications for cesarean delivery were almost equally distributed among 4 major factors: fetal distress; twinning; contraindication for induction (usually cesarean section); and uncorrected functional dystocia.

Among the 159 cases of cesarean section, the indication for interruption was preterm labor in 63 cases, fetal distress in 19 cases, chorioamnionitis in 10 cases and PPROM at 36 weeks in 59 cases. Among the 161 cases where the indication for resolution was preterm labor, caesarean section occurred in 63 (39.13%), and the indications were as follows: twinning (19 cases); functional dystocia (9 cases); breech presentation (8 cases); fetal malformation (6 cases); fetal distress (5 cases); iterativity (4 cases); maternal pathology (3 cases); uterine myomatosis (2 cases); previous tumor (2 cases); contracted pelvis (2 cases); meconium (1 case); cephalopelvic disproportion (1 case); and HIV (1 case). Among the 79 cases that reached the 36^th^ week, 59 were submitted to cesarean delivery (rate of 74.68%), and the indications were as follows: contraindication to induction (16 cases); functional dystocia (11 cases); iterativity (6 cases); breech presentation (6 cases); twinning (5 cases); intrapartum fetal distress (4 cases); induction failure (3 cases); meconium (2 cases); maternal pathology (2 cases); cephalopelvic disproportion (2 cases); and HIV (2 cases). Of the total cases, 48 were submitted to induction of labor. Of these, 20 ended in cesarean delivery (rate of 41.66%). Among the 18 patients who developed chorioamnionitis, 10 underwent cesarean delivery (55.55%), with the following indications: contraindication to induction (4); fetal distress (2); twinning (1); breech presentation (1); anomalous presentation (1); and cord prolapse (1).

**Figure 4:**
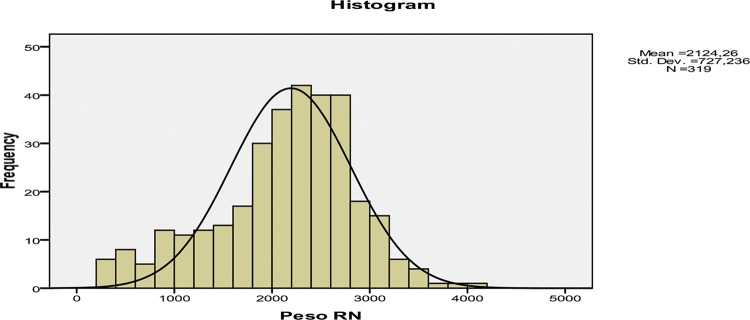
Distribution of the weight at birth, in grams, of 324 newborns of mothers with PPROM. Sao Paulo, Brazil, 2006-2011.

Among the 290 pregnant women, there were 324 newborns, of which 256 were singletons, and 68 were twins. Viability (GA ≥26 weeks) was reached by 301 of the fetuses (92.9%), and almost one-third of the infants (95, 29.32%) reached the 36^th^ week. However, at delivery, 111 newborns (34.26%) were less than 34 weeks of GA, and 48 (14.81%) were less than 30 weeks of GA. The birth weights varied between 280 and 4,170 grams, with the distribution presented in Figure 4.

There were 250 (78.6%) newborns with adequate weight for GA (AGA), and 65 (20.4%) were small for gestational age (SGA), according to the Alexander classification. The rate of low birth weight (<2,500 grams) was 67%; 18.9% of the sample weighed less than 1,500 grams, and 10.1% of the sample weighed less than 1,000 grams. There was a slightly higher prevalence of female newborns (50.78%) in relation to males (49.22%).

Approximately one-third of the newborns (117 or 36.9%) had a 1-minute Apgar<7. At 5 minutes, 48 newborns (15.1%) scored <7. Acidosis (pH<7.20) was present in the umbilical cords of 47 newborns (39.2%). The relatively small number of newborns with cord pH-metry is noteworthy. When checking the possible associated factors, we found a similar labor time (5.2 *vs*. 4.9 hours, *p*=0.576), but a lower mean GA at delivery (32.88 *vs*. 34.57 weeks, *p*=0.000) and a lower mean birth weight (2,027 *vs*. 2,337 grams; *p*=0.001) for the group without pH-metry compared to the group with pH-metry, indicating possible technical difficulties in sample collection. On the other hand, there were lower Apgar scores in the children without pH data, in the first minute (6.53 *vs*. 7.71, *p*=0.005), the fifth minute (7.81 *vs*. 9.04, *p*=0.008) and even at the tenth minute (8.17 *vs*. 9.41; *p*=0.011), suggesting perhaps a greater urgency for care.

The analysis in Table 4 presents the numerical variables for the newborns.

Two hundred eighty-four babies (89% of the sample) were born alive. Seventeen cases of fetal death occurred, for a stillbirth rate of 5.33%. Eighteen cases of neonatal death were reported, for a neonatal mortality rate of 5.64%. Thus, the perinatal death rate was 35/319=10.97%.

There were complications in childbirth and postpartum in 40 cases (18%), which were distributed as follows: 14 cases of vaginal and/or cervical lacerations; 4 cases of placental retention; 3 cases of uterine hypotonia/atony; 3 cases of increased bleeding; and 2 cases of focal placenta accreta. Isolated cases of hemoamnion, placental incarceration, ultimate head in pelvic delivery, corporal cesarean section, laceration of the uterine segment, cord prolapse, and even cord rupture were also described. Six patients (2.9%) required blood transfusion, and 2 (0.9%) required vasoactive drugs. Endometritis was diagnosed in the puerperium of 7 patients (3.3%). Sepsis was diagnosed in 2 patients; in one patient, it progressed to shock. There were no cases of hysterectomy or maternal death. Clinically, the patient’s general condition was regular in the puerperium on 7 occasions (2.4%), with malaise and a visible change in clinical status. Fourteen patients (6.6%) required intensive observation in the intensive care unit (ICU) or postanesthetic care unit.

## DISCUSSION

Because this study was performed at a tertiary hospital with a large number of patients with comorbidities and a protocol that leads to pregnancy up to 36 weeks, it differs somewhat from most other national or international studies. Maternal age is one potential area of difference; with an average of 27.5 years, the maternal age of our patients was similar to that reported in another hospital in the metropolitan region of São Paulo among women <28 weeks pregnant, which was 27.2 years (8). However, the average maternal age of our subjects was higher than that reported in other Brazilian studies; those values were 25.4 years in Acre (10), 25.7 years in Recife (9), 26.0 years in Santa Catarina (17), and 26.7 years in Rio de Janeiro (7). Maternal age was also higher than that reported in a Peruvian survey, 26.36 years (18). In contrast, our patients had a lower average maternal age than those reported in Pakistan, 30 years (19), Canada, 28.3 years (20), and a multicenter American study, 29.7 years (21). Such findings are in agreement with the report of a Brazilian study in Rio Grande do Sul (22), which accounted for a 2.49-fold higher risk of PPROM among pregnant women aged >30 years. Higher maternal age could be related to the more frequent presence of maternal diseases, such as hypertension and diabetes, or obstetric pathologies, such as twinning and fetal malformations.

However, the mean GA on admission, which was 31.89 weeks (with a median of 34 weeks), appears more compatible with the data in the literature. Our findings were similar to the GA described in 86 pregnant women from Recife-PE, in the northeastern region of Brazil, 31.9 weeks (12). However, in the latter study, patients with comorbidities were excluded, and only women between 24 and 35 weeks of gestation were included. In addition, our findings differ from others, such as those from a hospital in ABC Paulista, a metropolitan region of São Paulo (8), at 21.7 weeks (which included only patients with a GA <28 weeks), and those from a hospital in Acre, in the northern region of Brazil (10), at 34.7 weeks (including term and preterm pregnant women).

Regarding ethnicity, we observed a higher frequency of PPROM in white pregnant women (57.9%) than in black women (6%). This finding was similar to data from Rio Grande do Sul, with a frequency of 69.9% among white women (22), and the region of ABC Paulista, with 60.6% (8), but was discordant with some American data, which showed a greater risk among black women. In fact, a population cohort of more than 600,000 births in Missouri (23) showed a higher risk of PPROM among black women than among white women, with an OR of 2.3 (95% CI: 2.0-2.5). The risk was even greater for PPROM occurring before 28 weeks of pregnancy, with an OR of 2.8 (95% CI: 2.5-3.2). However, another American multicenter study (21) pointed to a predominance of PPROM among white pregnant women (55%).

The education level in our sample was relatively high; the majority of the patients (57.4%) had an education above elementary school, and 5.4% were university students. Such data are contrary to a large proportion of the literature, which establishes a higher risk of PPROM among women with less schooling (10,22,24).

Regarding marital status, most of the patients in the present study were in a consensual union (43.5%) or were single (28.4%), and slightly more than one-quarter of them were married. In comparison, in Acre (10), the rate of single women was very similar (28.7%), but in Fortaleza-Ceará, in the Brazilian northeast (11), the rate of single women was twice as high (66%).

Considering occupation, almost half of the patients analyzed (44.1%) described themselves as housewives (or as domestic workers). This rate may appear high, but it is lower than the rates reported by other authors in Fortaleza-Ceará, in the Brazilian northeast (11), of 49.7%, or in Cuba (25), of 71.4%. The latter study found among the housewives a 1.5-fold higher relative risk of PROM after the 30^th^ week of pregnancy.

Almost all of the patients in this study received prenatal care, either at our service (56%) or externally (39%), with a mean number of consultations (six) that was adequate for the duration of gestation, possibly because of the relatively early onset of prenatal care (mean GA of 15.69). These data differed, for example, from the Peruvian situation (18), where little prenatal control was reported (mean of 2 visits, and 26.7% of the sample did not receive prenatal care).

An important characteristic of our work was the large number of patients with some comorbidity (69.2%), which gives it a very specific and unusual profile compared with other studies because most studies exclude patients with comorbidities (7,8,9,11,19). Because this was an epidemiological study, we considered it important to include such patients to obtain a better notion of the daily reality of our hospital, which may be similar to that of others.

Because ours is a tertiary hospital with national referrals in a state with great migratory flow, it is not surprising that almost one-third of the patients (31.4%) were from other states or even other countries, which also distinguishes our sample from those of other studies.

The mean BMI measured at admission (29.1 kg/m^2^) or reported at the first prenatal visit (27.5 kg/m^2^) revealed a group of pregnant women who were mildly overweight. This finding is in line with those of a Chinese study (26) that included more than two thousand pregnant women and found a greater risk of PPROM among those with pregravid overweight. In this study, the PROM rate was progressively higher with higher pregravid BMI. A study conducted in the Brazilian northeast (9) reported a mean BMI very similar to that reported in our work (27.1 kg/m^2^), but it excluded patients with hypertension, diabetes and other clinical diseases.

The frequency of fetal malformations in our population (13.4%) was relatively high. Another Brazilian study (9) reported 5 malformations among 159 pregnant women (3.14%), all of whom were excluded from the final analysis. We believe that this rate was high in our sample because of the tertiary profile of the Hospital das Clinicas, which is a reference center for malformations. In fact, the correlation between congenital abnormalities per se and PPROM has not been sufficiently investigated as a result of the exclusion of such abnormalities in almost all publications on PPROM (7,8,9,11,19,20).

Twinning is also routinely excluded from research on PROM. A Thai study (27) reported statistically similar PROM rates for single and multiple pregnancies, although there was a lower trend among twin pregnancies (5.4%) than among other pregnancies (9%). However, when we considered the data of some authors (28) indicating that twinning is present in 2 to 3% of all pregnant women and constitutes at least 10% of preterm births, the rate that we identified (11.7%) was highly significant.

In our patients with PPROM, 8.1% were diagnosed with diabetes, a rate that appears high but that was lower than the 17.8% rate of gestational diabetes described at the same service (29) at another time, using new diagnostic criteria. In contrast, a Brazilian study that included 50 patients with gestational diabetes treated at a public maternity service in Ceará, northeastern Brazil, described an overall amniorrhexis rate of 16% in such patients, higher than the classically reported rate of 10% (30). Another study from Austria (31) reported a lower PROM rate followed by spontaneous labor in pregnant women with diabetes (41.6%) than in the control group (65.7%). It is possible that this correlation between diabetes and PROM is related to glycemic control because, as a study in Spain (32) established, patients whose gestational diabetes was discovered later presented with higher rates of polyhydramnios (12.7% *vs*. 2.1%) and PPROM (2.1% *vs*. 0%) than those whose gestational diabetes was discovered earlier.

Our rates of cervical incompetence and cerclage were relatively high (3.9% and 4.3%, respectively) considering that the rate of cervical incompetence in the general population varies between 0.1 and 1.8% (33). However, this finding was not surprising because PPROM is described as a major complication of cerclage in cases of cervical incompetence and occurred in 10.5% of cases in a Brazilian study based in Campinas (34). Additionally, a case-control study in the United States (35) showed that patients with PPROM had a higher frequency of cervical incompetence (6.9%) than patients in the control group (1.9%), OR=3.8 (95% CI: 1.2-11.6). This risk was even higher when cervical incompetence was present in the current pregnancy (14.7% x 1.0% - OR: 18.0; 95% CI: 5.1-63.6) and when the cerclage procedure had been performed (11.8% x 1.0% - OR=13.9, 95% CI, 3.8-50.4).

We had a relatively high rate of prior abortion: 32.3% of the sample had at least one abortion. Some authors (35) have described a history of abortion or pregnancy loss up to 20 weeks in 52.9% of their patients with PPROM, which clearly differed from that of the control group (33.5%). Other authors have described an association between PPROM and repeated abortions. Israeli authors (36), for example, reported a relative risk of 1.2 (95% CI: 1.1-1.3) for PPROM among patients with repeat abortions, with an incidence of 6.5% *vs*. 5.6% in the control population. Another Israeli study (37) reported even higher rates of repeat abortion among those with PPROM (9.4%), surpassing the prematurity group; in comparison, the rate among those without PPROM was 6.9%. In comparison, an American study (38) described increasing rates of PPROM according to the previous history of fetal loss: the rate was 2.6% in the absence of an antecedent, 3.2% when there was only one loss, 5.1% when there were two losses and 6.7% when there were 3 or more losses.

We also found high personal background rates of preterm birth (17.3%) and previous PPROM (6.6%) that were clearly higher than rates for the general population, but not as high as those reported in one American study (35), in which 26.6% experienced preterm delivery in the second trimester (OR 16.1), and 22.5% experienced preterm delivery in the third trimester (OR=3.2). These American authors also established a rate of 11.8% for previous PPROM; this rate was higher than the rate of 1.6% reported for the control population, thus yielding an OR=8.3. However, our rates are still higher than those indicated by a survey carried out in the Brazilian northeast (11), where 5.4% of pregnant women with PPROM had a history of PPROM, and 4.8% had previous prematurity. A classic study on the subject (39) previously indicated that previous premature birth was an important risk factor for PPROM, with OR=2.5.

We found a history of genital bleeding in the first half of pregnancy in 5.8% of the patients, a rate that may have been underestimated given the retrospective nature of this study but was otherwise significant. Some authors have presented higher rates, such as 9.8% (35), and others have reported lower rates (1.3%), but with an increased risk compared with the control group: OR 2.44 (37). Some studies have even cited a 7-fold increased risk for PPROM when genital bleeding is present (39).

The rate of smoking (14%) in the sample was also significant. It was higher than that reported in some Brazilian studies of smoking prevalence in pregnant women, such as the 4.1% rate reported for women in São Luis, Maranhão, in northern Brazil (40). Although some more recent authors (41) have not been able to identify a difference in the smoking rate between pregnant women with PPROM (28.9%) and those without it (26.84%), most authors assume an association between smoking and PROM, with at least double the risk of PROM among smokers (35,39,42,43).

Few studies have adequately described the initial clinical presentation of and the diagnostic methods for PPROM. We will compare our data with those of some Brazilian studies. The first study, conducted in Ribeirão Preto-SP in the southeastern part of the country (44), reported that fluid loss was present in 42% of the 107 cases evaluated, while we described it as a typical flow of liquid in 78.2% of the cases. Another study (45) of 29 pregnant women with PPROM at <26 weeks stated that the diagnosis was clinically confirmed by specular examination in 62.1% of the cases (in our study, clinical confirmation occurred in 83.3% of cases), and 31% had concomitant vaginal bleeding (in our data, this sign was only present in 12.7% of pregnant women). A study from Rio de Janeiro (7) reported that the diagnosis was confirmed by vaginal pH analysis using nitrazine paper and by observing arboriform crystallization of the liquid collected in the vaginal sac fundus, but it did not establish the rates of use of such procedures. In fact, most international studies also fail to describe in detail how the diagnosis was made. In this sense, the present study, which describes in detail how PPROM was diagnosed, has some relevance. It is known that PROM is easier to diagnose at term, when a large amount of amniotic fluid can be observed on pelvic examination. However, the lower the GA, the greater the difficulty of establishing the diagnosis because the amount of amniotic fluid that is externalized is lower. In our series, there was diagnostic doubt in 17.6% of the cases, and even the phenol test did not confirm the diagnosis in approximately 10% of the analyzed pregnant women. That is, diagnosis is difficult in clinical practice in many situations, which may lead to an increase in infection risk for the maternal-fetal pair. This situation indicates a gap that needs to be filled with more reliable tests.

Leukocytes - In the present study, the mean leukocyte count observed at admission was 11,173/mm^3^, with 9.5% leukocytosis. These rates are lower than those reported by the majority of authors (17,44,46,47) but higher than some reports (48).

CRP - In the present study, CRP presented a mean initial value of 15.96 mg/L, which is very close to the value of 14.80 mg/L reported in a Turkish study (46) but higher than the 8.37 mg/L at entry reported in an Italian study (47).

Chorioamnionitis - Our described rate of 10.8% for prepartum clinical chorioamnionitis is relatively low, even considering the final chorioamnionitis rate of 22.9%. In fact, most of the papers analyzed reported higher rates: 23.9% (18), 34.7% (9), 35% (46), 47% (8), 53% (47), and 67.1% (7). However, there were also studies with comparatively lower rates: 20.2% (49); 17.4% (21); 16.5% (37), 14.6% (10); 12% (19); 9% (20); and 2.4% (11). However, in the latter case (11), only 29.7% of the sample had PPROM, and the great majority were >37 weeks of gestation. In addition, 18.8% of the newborns were diagnosed with infection, which suggests that the actual rate of maternal infection must have been higher.

AFI - Our mean initial AFI was 8.1 cm, higher than the initial averages reported by some Brazilian authors: 3.9 cm (8) and 3.7 cm (12). A total of 27.9% of our patients had oligohydramnios at the initial evaluation, which was also lower than the rates reported by the majority of the authors analyzed [75.8% (45), 62.3% (46), 60.8% (9), 52.7% (50)] but higher than those reported in articles from other countries [China, 12.8% (51); and Israel, 4.3% (37)]. However, in China, only 11.2% of the cases were born preterm; in Israel, most cases (80.8%) were born between 33 and 36 weeks.

Vaginal Infections - Our Group B streptococcus detection rate (20.2%) was lower than that reported in southeast Brazil, in Campinas (52), 30%, but higher than the 14.8% reported in Spain (53). A rate of 14.8% was also described in another Brazilian article (54), but with normally progressing women in the third trimester of pregnancy, without rupture of membranes. A recent Chinese case-control article (55) identified Group B streptococcal infection as a risk factor for PROM, with rates of 22.3% in cases and 6.5% in controls. Interestingly, this rate was quite similar to our data. We also identified a 30% rate of bacterial vaginosis in our PPROM patients. This rate is similar to that presented by Nigerian authors for pregnant women with term PROM (56): 29.1%. However, our rate was substantially lower than that found by Japanese authors (57) in pregnant women with preterm labor who eventually evolved to PPROM: 72.9%.

Corticoids - The rate of corticosteroid use in this sample was close to 10%. Despite the wide variation among various authors, our rate is among the lowest; other studies have reported rates of 98% (9), 94.45% (17), 62.8% (25), 51.5% (8), 45% (21), 33.3% (10), 18.8% (11), 18.7% (44), and 6.9% (45). In contrast, a Canadian study (20) showed variable rates of corticosteroid use depending on the latency period, ranging from 31% (latency <48 hours) to 68.5% (latency ≥7 days).

Latency - The latency period averaged 9.17 days, with a duration of ≥3 days in almost half of the cases. Of course, this variable depends greatly on the population studied and the hospital’s protocol. If we had excluded patients with twin gestation, malformed fetuses or comorbidities, the latency period would certainly have been greater. The GA at diagnosis is also very relevant. A Brazilian study (11) in which the majority of cases were above 37 weeks of gestation had an average latency period of only 42 hours, with durations of ≤24 hours in 65.5% of the sample. In comparison, cases of PPROM before viability had a longer latency period, from 21.7 days (45) to 12 days (8); the latter paper reported a latency period <7 in only 7.6% of the cases. A Brazilian study from Santa Catarina, in the south of Brazil (17), with a slightly longer GA (20 to 33 weeks) reported a mean latency time of 12 days, with 54.5% of cases surpassing 7 days of latency. Canadian researchers (49) also reported an average latency of 12 days in patients hospitalized for PPROM. In studies that included a sample similar to ours, at least in terms of GA, the mean latency periods were similar: 10.5 days (9) and 10.68 days (18). A Brazilian study from Rio de Janeiro (7) reported a latency period of >48 hours in 66.5% of cases, which was slightly better than our results. The results of one Italian study (47) were also better, with an average latency period of 16 days. However, several authors reported worse data: a mean latency of approximately 3 days (46) and a latency period <24 hours in 69.2% of the sample, even when the GA at rupture was between 21 and 37 weeks (44). A Canadian study (20) presented similar results, with median latency periods of 87.6 hours (3.6 days) for the group with a GA between 24 and 34 weeks and 22.8 hours in the group with a GA between 34 and 36 weeks.

Our rate of urinary tract infection (UTI) during pregnancy was 13.7%. A Brazilian article from the Amazon region (58) that included 100 puerperae with preterm births had a PPROM rate of 15.6%, and 21.8% had clinically diagnosed UTI during prenatal care. Another national article, from Rio Grande do Sul (22), did not perceive an increased risk of UTI in pregnant women with PPROM compared with control patients; the rates were 3.4% in the former and 2.9% in the latter. However, other case-control studies observed significant differences. One of them, from India (59), described a history of UTI in 6% of patients with PROM and in 2% of control patients, with OR=3.5 (1.05-14.9). Another study from Cuba (60) found a significant relative risk of 1.33 (1.15-1.53), with a UTI rate of 14.7%, very similar to ours. A third study, from Israel (37), also found an increased risk (OR=1.55) of UTI in patients with PPROM (5.1%) compared with other patients with preterm delivery but without PROM (3.3%).

Type of delivery - We had a cesarean rate of 55%, which is the average rate at our institution in recent years. These numbers are similar to the cesarean rates described in works from Italy (47), with 57.5%; the United States (21), with 57.4%; Iran (50), with 56%; Cuba (25), with 48.5%; and Canada (49), with 48.3%. Some studies (11,18) show higher cesarean rates of close to 72-74%. In contrast, some authors (7,8,9,20,24,42,44) had lower cesarean delivery rates, ranging from 14 to 39%. In view of the previously described conditions of our population, we consider our cesarean rate adequate and comparable to that of other authors in similar situations. It is worth noting that our cesarean rate was due in large part to the presence of previous uterine scarring, which in our service contraindicates the induction of labor. In cases with spontaneous labor, the cesarean rate was only 39%, the main indications were twinning and pelvic presentation. In the cases that reached the 36^th^ week, the cesarean rate was higher (almost 75%), the main indications being contraindication to induction (usually the presence of an anterior cesarean section), functional dystocia and iterativity (two or more cesareans). That is, our very high cesarean rate is related not only to the PPROM itself, but also to the demographic characteristic of our population, with high rates of previous cesarean section.

Indication for delivery - The main indications for the cesarean sections performed in our sample were fetal distress, contraindications for induction (usually because of previous cesarean section), functional dystocia and pelvic presentation, excluding cases of twinning and fetal malformation. It is difficult to compare other studies with ours because few authors have clarified the reasons for selecting a specific type of delivery and because differences in obstetric protocol hamper interpretation. The presence of comorbidities in more than half of our sample raises the risk of fetal distress, which was not reported in other studies. However, we can comparatively analyze some studies. One study (47) described similar indications, with changes in cardiotocography (52.6%), followed by noncephalic fetal presentation (21%). The latter was the main indication reported by another group of investigators (8) for the upper route (42.4%), followed by previous cesarean section and fetal distress (19.2% each). Ina Cuban survey (25), the main indication was induction failure (35.1%), followed by anhydramnios and ovular infection (23.6% each). Similarly, signs of intrauterine infection were the main reason for cesarean section reported by other authors (44); such signs occurred in 40.9% of cases.

Induction of labor - Certainly, maintaining the pregnancy until the 36^th^ week increases the chance of spontaneous labor (which was present in 55% of our patients) and reduces the rate of resolution by the induction of labor, which occurred in 16.4% of our sample. Virtually all other authors reported higher rates of induction: 22.7% (8); 28.6% (25); 32.4% (9); and 62.6% (44). The only perceived exception was an Israeli study (37) of pregnant women with PPROM up to 36 weeks, which described an 8% rate of induction with oxytocin and a 3% rate of induction with vaginal prostaglandin. These rates were higher than those for the control group of pregnant women who had preterm birth without PROM: 3.3% and 6.1%, respectively.

Intercurrences at delivery and postpartum - We noted some intercurrences at delivery and postpartum in 18% of the sample, most of which were routine intercurrences, such as laceration of the birth canal. If we did not consider this occurrence, we would have had a lower rate of 8.9%. The main intercurrences were endometritis (3.3%); bleeding during the 3^rd^ and 4^th^ periods (3.1%), which required blood transfusion in 2.9% of cases; and a 0.7% rate of maternal sepsis. Our data are better than most reports in the literature. We only identified one Israeli study (37) with a lower rate of endometritis (2.8%) than ours, but this rate was higher than that of the control group, which had preterm delivery but without PROM (1.4%). However, these authors described other complications during childbirth, such as placental retention requiring the manual removal of the placenta (2.1%), wall infection (2.1%) and bacteremia (9.4%), which resulted in an overall complication rate of 16.4%. In Peru (18), a 10.6% rate of infection at the operative site, a 2.1% rate of maternal sepsis, and a 2.8% rate of *abruptio placentae* with bleeding were described, while in Cuba (25), an endometritis rate of 8.57% was reported. In comparison, in Pakistan (24), the authors reported a rate of 16.5% for infectious complications. In Canada (49), some maternal morbidity was described in 59.6% of patients hospitalized for PPROM, with a 22.7% rate of antepartum hemorrhage. In Iran (50), a 16.5% rate of hemorrhagic complications at delivery was reported. In Brazil, authors (45) have reported a 17.2% rate of postpartum infectious disease, in addition to a 3.4% rate of postpartum hemorrhage, while others (44) described only a 1.8% rate of postpartum endometritis but a 14.2% rate of fever in the immediate puerperium, in addition to one maternal death (0.93%). We believe that the comparatively lower rate of puerperal complications in our data was associated with our hospital’s policy of expectant management, which involved lower rates of induction and operative delivery. This reasoning is supported by the findings of a Dutch research group (61), where patients who underwent active management showed a tendency toward an increased risk of maternal sepsis and a reduced risk of neonatal sepsis, with a significant increase in the rate of neonatal fever, compared with patients treated with expectant management. A similar pattern can be observed in study data from a Pennsylvania hospital (62), which, by changing the service protocol so that the resolution of PPROM cases was anticipated at 34 weeks rather than 36 weeks, observed increased rates of chorioamnionitis (from 3.4 to 8%), endometritis (from 0 to 4%) and cesarean section (from 20.7 to 44%).

Days of hospitalization - In our sample, the mean number of days of maternal hospitalization was 9.18 days; it was ≥7 in 40.2% of cases. This average was lower than that reported by the majority of the other authors: 13.6 days (9); 15.3 days (18); and 19.1 days (8). A Canadian study (49) that compared hospital care with home care reported an average length of hospital stay of 14 days for the hospital group.

Birth weight - Our data indicated a mean birth weight of 2,124 grams; 67% of newborns were low birth weight (LBW), 18.9% weighed less than 1,500 grams (very low birth weight - VLBW), and 20.4% of the sample was SGA. Comparisons with other reports highlight our good results. An exception was a Pakistani study (24), which reported better indexes than ours, including an LBW rate of 62.3% and a VLBW rate of 11.76%. A multicenter Chinese study (51) that included newborns between 26 and 36 weeks GA indicated a mean birth weight similar to that of our study but slightly lower; the mean was 1,997 grams, but the sample showed wide variation (ranging from 700 to 3,900 grams). Other studies had worse numbers, such as a recent Iranian study (50) that indicated mean birth weights of 1,730 grams in the normal AFI group and 1575 grams in the group with oligohydramnios. A recent Canadian study (49) reported a median weight of 1,807 grams for infants born to patients hospitalized for PPROM, but only 4.5% of the infants showed fetal weight restriction. Three studies reported mean birth weights lower than ours but included GAs up to 34 weeks: 1,653 grams (18), 1,725 grams (17), and 1,483 grams (7). A Cuban study (25) that also included patients with PPROM up to 34 weeks reported that all newborns were underweight (<2,500 g), and 47.3% weighed <1,500 grams. A study from the Brazilian northeast (9) reported follow-up data up to 35 weeks, with an average birth weight of 1,711 grams and a high LBW rate of 90.2%. In comparison, Israeli research (37) that included 968 pregnant women up to 36 weeks reported that 66.7% of newborns weighed ≤2,500 grams, but only 3.4% had fetal growth restriction. Other studies described a case-series of patients with PPROM before viability and showed an even more unfavorable picture: an average weight of 1,324 grams, with an 87.8% rate of LWB (8) and a 17.2% rate of SGA (45).

GA at delivery averaged 33.49 weeks, with 38.9% of patients reaching 36 weeks. This good result was related to the casuistry studied, which described cases up to the limit of late prematurity. Few studies have focused on this broader GA profile. A multicenter Chinese study (51) is one of the few studies presenting PPROM results up to 36 weeks; it reported a mean GA at delivery of 32.4 weeks, ranging from 24 to 36 weeks. Other studies have not evaluated late prematurity; therefore, they had lower GAs at birth: 32.3 weeks (46); 31.7 weeks (7); 28.4 weeks (8).

Apgar scores - Our data indicate that 31.8% of newborns had 1-min Apgar scores <7, while only 11.2% had 5-min Apgar scores <7. Again, our data demonstrate better results than those of most authors, possibly because of the higher GA of our sample. Studies of participants with higher GA at delivery had better rates: 1-min Apgar score <5 in 12.3% and 5-min Apgar score <7 in 3.8% (37); 5-min Apgar scores ≤7 in 4.8% of newborns (11); 1-min Apgar scores <7 in 34.5% and 5-min Apgar scores <7 in 9.8% (18). An American study (21) that included pregnant women with GA <32 weeks and excluded twins reported intermediate rates: 7.7% had 5-min Apgar scores <5. A Canadian study that included more than four thousand pregnant women with PPROM (20) presented two different rates for Apgar scores: when the GA was between 24 and 34 weeks, 5-min Apgar scores <7 occurred in 6.7% of patients, while for GAs between 34 and 36 weeks, the rate was only 1.4%. Other authors whose samples had a lower GA described more worrisome numbers: 1-min Apgar scores <7 in 51% of the newborns and 5-min Apgar scores <7 in 12.2% (8); 5-min Apgar scores <7 in 17.9% (7); 1-min Apgar scores <7 in 37.9% (25) and low 1-min Apgar scores in 30.5% (24); and finally, 1-min Apgar scores with a median of 7 (interquartile range 4-9) and 5-min Apgar scores with a median of 9 (interquartile range 8-9) (9).

Perinatal mortality - For our entire series, which included malformed and twin fetuses, we obtained a stillbirth rate of 5.33%, which, together with the neonatal mortality rate of 5.64%, yields a perinatal mortality rate of 10.97%. If, like most authors, we had excluded these higher-risk subgroups, we would have had better indexes (3.7% neonatal mortality, 4.2% stillborn mortality, and 7.9% perinatal mortality). In any case, compared with the results of other authors, our results appear favorable. While a Cuban study (25) had better rates (2.63%) for stillbirths and the same rate of neonatal mortality, for a total perinatal mortality rate of 5.26%, other studies computed worse rates. An American study (21) reported 3.22% stillbirths (half of them in the delivery room) and 7.1% neonatal mortality, for a total perinatal death rate of 10.32%, but this study excluded twin pregnancies and births at >32 weeks. An Israeli study (37) with a population similar to ours that included pregnant women up to 36 weeks of gestation reported a fetal mortality rate of 1.75% (including an 0.8% rate of intrapartum death) and a neonatal death rate of 5.5%, resulting in a perinatal mortality rate of 7.23%. A Pakistani study (24) reported a fetal death rate of 5.8% and a neonatal death rate of 12.9%, yielding a perinatal death rate of 18.8%. An Iranian study (50) reported a neonatal death rate of 14.3%, which was significantly higher when the AFI was <5, with an RR=6. Brazilian studies have also reported discouraging indexes; the exception was one study that had a predominance of patients close to term (11) and reported a neonatal death rate of only 1.2%. The others presented the following results: 25.7% fetal deaths and 16.7% neonatal deaths, totaling 42.4% (8); neonatal deaths in 14.45% (7); a stillbirth rate of 4.54% and a neonatal mortality rate of 13.63%, totaling 18.17% (17); and 10.3% fetal deaths and 86.2% neonatal deaths, with a very high perinatal mortality rate of 96.5% (45). To better illustrate the important influence of weight and GA on perinatal mortality, one Brazilian study (9) reported a total perinatal mortality rate of 29%, which reached 100% when the newborn’s weight was ≤1,000 grams and zero when the weight was >2,500 grams.

## AUTHOR CONTRIBUTIONS

Galletta MAK was responsible for the study conceptualization and design, data acquisition, analysis and interpretation, investigation, manuscript writing, original draft and review, and statistical analysis. Bittar RE was responsible for the study conceptualization and design, manuscript writing and review, and administrative support. Agra I and Guerra ECL were responsible for the data acquisition. Francisco RPV was responsible for manuscript writing and review, administrative support, and supervision. Zugaib M was responsible for administrative support and supervision.

## Figures and Tables

**Table 1:** Characterization of the sample of 299 pregnant women with PPROM, with numerical variables.

Variable	Mean	Median	Variation (min-max)	25%-75%	Standard deviation	n
Age (years)	27.46	27.00	14-44	23-32	6.591	299
Initial weight at PNC (kg)	69.73	66.20	44.85-150.0	57.25-77.70	17.67	120
Weight at admission (kg)	73.91	71.70	47.0-154.7	64.10-79.00	15.26	216
Initial BMI at PNC	27.50	26.17	15.33-56.45	22.99-30.73	6.486	116
BMI at admission	29.12	28.10	18.46-58.22	25.32-31.99	5.792	189
Initial GA at PNC	15.69	14.00	4.0-34.0	10-20	6.933	247
GA at diagnosis	31.89	34.00	8.0-36.85	30.85-35.41	5.277	295
GA at admission	32.16	34.14	14.28-36.85	31.00-35.42	4.913	299
Number of prenatal visits	6.01	6.00	0-19	4-8	2.961	259

Abbreviations: GA = gestational age; PNC = prenatal care; BMI = body mass index (kg/m^2^); n = number. Sao Paulo, Brazil, 2006-2011.

**Table 2:** Categorical variables characterizing the sample of 299 pregnant women with PPROM.

Variable	Number of Cases	Category 1	Category 2	Category 3	Category 4
Color	299	White: 173 (57.9%)	Brown: 105 (35.1%)	Black: 18 (6.0%)	Yellow: 3 (1.0%)
Gesta	298	1G: 102 (34.2%)	2G: 67 (22.5%)	3G: 46 (15.4%)	4G: 39 (13.1%)
Para	298	0P: 130 (43.6%)	1P: 74 (24.8%)	2P: 49 (16.4%)	3P: 29 (9.7%)
Abortion	298	0A: 201 (67.7%)	1A: 62 (20.9%)	2A: 18 (6.1%)	3A: 7 (2.4%)
Place of birth	299	SP: 205 (68.6%)	BA: 26 (8.7%)	MG: 12 (4.0%)	PE: 10 (3.3%)
Education level	299	2^nd^ DC: 109 (36.8%)	1^st^ DC: 58 (19.6%)	1^st^ DI: 50 (16.9%)	2^nd^ DI: 45 (15.2%)
Marital status	299	Consensus union: 130 (43.5%)	Single: 85 (28.4%)	Married: 78 (26.1%)	Divorced: 3 (1%)
Occupation	297	Domestic worker: 132 (44.1%)	Student: 27 (9.0%)	Housemaid: 17 (5.7%)	Salesperson: 12 (4.0%)
Local of PNC	298	PNC at HC: 167 (56.0%)	Outside HC: 117 (39.3%)	No PNC: 14 (4.7%)	

Abbreviations: 1^st^ DI: first degree incomplete; 1^st^ DC: first degree complete; 2^nd^ DI: second degree incomplete; 2^nd^ DC: second degree complete; SP: São Paulo; BA: Bahia; MG: Minas Gerais; PE: Pernambuco; PNC = prenatal care. Sao Paulo, Brazil, 2006-2011.

**Table 3:** Evolutionary data of 290 pregnant women with delivery in the institution.

	Statistics							
	N		Mean	Median	Std. Deviation	Percentiles		
	Valid	Missing				25	50	75
Initial AFI	146	144	8.180	7.300	6.2819	4.575	7.300	10.350
Final AFI	128	162	6.865	6.500	4.4817	3.925	6.500	9.275
Initial Leuko	163	127	11,126.75	10,760.00	3,461.167	9,020.00	10,760.00	12,500.00
Final Leuko	203	87	12,230.10	11,380.00	4,485.653	9,330.00	11,380.00	14,230.00
Initial CRP	90	200	15.32	7.64	21.669	3.75	7.64	15.52
Final CRP	146	144	27.11	10.10	39.916	4.52	10.10	21.90

AFI = Amniotic Liquid Index; Leuko = Leukocyte count; CRP = C Reactive Protein.

**Table 4:** Numerical variables related to birth of 324 newborns of mothers with PPROM.

Variable	Mean	Median	Variation (min-max)	25%-75%	Standard deviation	n
GA at birth	33.49	34.93	20.81 to 36.86	32.45 to 36.00	3.665	324
Weight at birth	2,124.2	2,240	280 to 4,170	1,750 a 2,640	727.23	319
Apgar 1 min	6.93	8.00	0 to 10	6 to 9	2.845	317
Apgar 5 min	8.29	9.00	0 to 10	8.5 to10	2.616	317
Apgar 10 min	8.67	10.00	0 to 10	9 to 10	2.648	315
Cord pH	7.216	7.226	6.800 to 7.388	7.167 to 7.279	0.094	120

GA = gestational age. Sao Paulo, Brazil, 2006-2011.
